# Mapping Uncertainty Due to Missing Data in the Global Ocean Health Index

**DOI:** 10.1371/journal.pone.0160377

**Published:** 2016-08-02

**Authors:** Melanie Frazier, Catherine Longo, Benjamin S. Halpern

**Affiliations:** 1 National Center for Ecological Analysis and Synthesis, University of California Santa Barbara, Santa Barbara, California, United States of America; 2 Marine Stewardship Council, 1 Snow Hill, London, EC1A 2DH, United Kingdom; 3 Silwood Park Campus, Imperial College London, Ascot, West Berkshire, United Kingdom; 4 Bren School of Environmental Science and Management, University of California Santa Barbara, Santa Barbara, California, United States of America; National Oceanic and Atmospheric Administration, UNITED STATES

## Abstract

Indicators are increasingly used to measure environmental systems; however, they are often criticized for failing to measure and describe uncertainty. Uncertainty is particularly difficult to evaluate and communicate in the case of composite indicators which aggregate many indicators of ecosystem condition. One of the ongoing goals of the Ocean Health Index (OHI) has been to improve our approach to dealing with missing data, which is a major source of uncertainty. Here we: (1) quantify the potential influence of gapfilled data on index scores from the 2015 global OHI assessment; (2) develop effective methods of tracking, quantifying, and communicating this information; and (3) provide general guidance for implementing gapfilling procedures for existing and emerging indicators, including regional OHI assessments. For the overall OHI global index score, the percent contribution of gapfilled data was relatively small (18.5%); however, it varied substantially among regions and goals. In general, smaller territorial jurisdictions and the food provision and tourism and recreation goals required the most gapfilling. We found the best approach for managing gapfilled data was to mirror the general framework used to organize, calculate, and communicate the Index data and scores. Quantifying gapfilling provides a measure of the reliability of the scores for different regions and components of an indicator. Importantly, this information highlights the importance of the underlying datasets used to calculate composite indicators and can inform and incentivize future data collection.

## Introduction

Given increasing human demands on natural systems [1–6], effective management is essential to maintaining a healthy environment that can sustainably deliver a range of benefits to people [7–10]. To be effective, management of natural systems requires measuring their condition and clearly communicating this information to a broad audience. To meet this need, there has been a proliferation of environmental indicators that distill large amounts of complex information into quantitative metrics that describe the condition of some aspect of our environment (e.g., biodiversity, water quality, etc.) [11–13]. The need to provide more comprehensive assessments of complex systems, such as marine ecosystems, has resulted in aggregating environmental indicators into composite indicators (or, indicator frameworks) [14–16]. Indicators, and particularly composite indicators, can be used to determine whether broad environmental objectives are being met, monitor trends in environmental condition, and communicate with the general public, scientists, resource managers, and policy makers [17–19]. Despite their utility, the underlying data and models used to calculate composite indicators can be of varying quality, consequently, the responsible use of indicators depends on transparent descriptions and estimates of uncertainty [15,20–22].

Indicators are mathematical models that represent a system’s condition and, as with any model, uncertainty is inevitable and can be introduced in many ways [15,23–25]. Uncertainty can result when the underlying data have large amounts of natural variation, measurement error, or missing values; or, when the indicator models fail to accurately estimate condition because they are inadequately parameterized, rely on proxy data that is a poor substitute for the information it replaces, or have flawed reference points used to rescale data. Furthermore, for composite indicators, the overall framework may fail to accurately represent an ecosystem if indicators are not well selected or the weights used to aggregate indicators do not reflect their relative importance in the system. Methods of quantifying uncertainty for composite indicators have been developed [3,15,20,21] and have been applied to some composite indicators [24,26,27].

A major source of uncertainty, related to data quality, arises from gapfilling, which is the process of estimating missing data to obtain as complete a dataset as possible [15]. A variety of methods are used to estimate missing data, including: averages of closely related groups (e.g., regions sharing ecological, spatial, political attributes; taxonomic groups; etc.), spatial or temporal interpolation (e.g., raster or time-series data), and predictive models (e.g., regression analysis, machine learning, etc.). In the case of a global composite index, gaps in data arise from variation in both data quality and availability. Many developing countries lack the resources to gather detailed datasets, and even developed, data-rich countries have inevitable data gaps. Given how common gaps in data are, clear documentation of gapfilling is a critical step of index development because it provides a measure of the reliability of index scores.

To address this issue, we demonstrate a transparent approach to gapfilling missing data and quantifying the contribution of gapfilled data to Ocean Health Index (OHI) scores. The OHI [28–30] measures the state of the world’s oceans, based on the performance of 10 goals that describe how people benefit from marine systems (Table 1). Goal scores are combined to obtain an overall index score for each region. Starting in 2012, the OHI has been assessed annually to obtain scores for 220 countries and territorial jurisdictions (referred to as ‘regions’). The global OHI assessment requires large amounts of data collected by a variety of institutions that are not always complete and vary in quality as well as spatial and temporal scale.

**Table 1:** The ten goals of the Ocean Health Index.

Food Provision	The sustainable harvest of seafood from wild-caught fisheries and mariculture
Artisanal Fishing Opportunity	The opportunity for small-scale fishers to supply catch for their families, members of their local communities, or sell in local markets
Natural Products	The natural resources that are sustainably extracted from living marine resources
Carbon Storage	The condition of coastal habitats that store and sequester atmospheric carbon
Coastal Livelihoods and Economies	Coastal and ocean-dependent livelihoods (job quantity and quality) and economies (revenues) produced by marine sectors
Tourism and Recreation	The value people have for experiencing and enjoying coastal areas through activities such as sailing, recreational fishing, beach-going, and bird watching
Sense of Place	The conservation status of iconic species (e.g., salmon, whales) and geographic locations that contribute to cultural identity
Clean Waters	The degree to which ocean regions are free of contaminants such as chemicals, eutrophication, harmful algal blooms, disease pathogens, and trash
Biodiversity	The conservation status of native marine species and key habitats that serve as a proxy for the suite of species that depend upon them

The OHI’s approach to dealing with missing data has evolved over time. The initial 2012 global OHI described the general methods used to gapfill missing data but did not attempt to track gapfilling across regions and goals [28]. The 2013 assessment [29] generally described which regions and goals used gapfilled data, but the analysis was limited to one type of gapfilling (*regional* gapfilling, see methods for descriptions) and one component of OHI scores (current status, but not pressure, resilience, or trend). Furthermore, gapfilling was only qualitatively described using broad categories (none, partial, full).

For the current OHI assessment, we substantially improved our approach to gapfilling. We transparently document how each of the underlying datasets was gapfilled, and we calculate the percent contribution of gapfilled data to each region’s scores for the 10 goals and the overall index. Although this does not provide a direct estimate of the uncertainty introduced by gapfilled values (i.e., we do not generate confidence intervals around scores), it offers a rapid, large-scale assessment of data-poor ‘hot spots’ by geographic area and goal. We also began implementing methodological improvements to gapfilling, such as the use of statistical methods (e.g., cross-validation) to select the best method of estimating missing data when there are multiple possible approaches and to better estimate the error associated with these estimates.

Although we focus on gapfilling in the context of the OHI, our methods are transferable to any index. We offer specific recommendations for tracking gapfilled data, selecting among gapfilling methods, and estimating error due to gapfilling. Quantifying the proportion of gapfilled data provides a measure of the reliability of scores for different regions and components of an indicator. This information improves the transparency of indicators and promotes their responsible use. Perhaps most importantly, this information highlights the importance of the underlying datasets used to calculate composite indicators and can inform and incentivize future data collection that is critical for measuring environmental systems.

## Materials and Methods

Calculating OHI scores requires many datasets (S1 Table) that, in addition to the region and the variable of interest, often include temporal data (e.g., year) and other information, such as commodity type or fisheries stock. Gapfilling typically occurs during the preparation of these data layers (S1 Table), although it can occur during subsequent steps in the calculations. The datasets are used in a series of models to calculate scores for 10 goals pertaining to how people interact with and benefit from marine systems. Each goal’s score is an average of its current status and projected future state. The current status compares the current condition of each goal to a defined reference point. The projected future state is an estimate of a goal’s condition after five years based on recent trends in status, as well as current levels of pressure and resilience conditions (see [28,29] for full details). The trend component of a goal’s score is the average yearly change in status, estimated from a linear regression of the five most recent years of status data, and then projected five years into the future (i.e., the slope is multiplied by five). The pressure score describes the cumulative pressures acting on a goal, resulting in an expected decrease in the goal score. The resilience score reflects the current ecological and social conditions expected to mitigate these pressures, such as food web integrity (ecological) and governance capabilities (social). After the 10 goal scores are calculated for each region, they are averaged to calculate an index score for each region. Finally, an overall global score is calculated as the EEZ area-weighted average of the regional scores.

The uncertainty introduced by gapfilling for each region’s 10 goal and index scores depends on: if data were gapfilled, the error introduced by gapfilling, and the relative contribution of a dataset to scores. We determined the percentage of each score that was calculated using gapfilled data, based on which values were gapfilled in each dataset and the percent contribution of each dataset to scores based on the OHI models (S1 File). For example, the status component of the artisanal fishing opportunities goal is calculated using two variables (economic need and access, S1 File) that have equal weight. The availability of data for these two variables differs among regions. If one of these two variables are gapfilled for a region, the percent contribution of gapfilled data to the status component of this goal would be 50% (the overall goal score would also factor in the percent contribution of gapfilled data for the trend, pressure, and resilience components). If both variables were gapfilled, the percent contribution of gapfilled data would be 100%. The percent contribution of gapfilled data informs us about the potential uncertainty being introduced. However, because we do not incorporate estimates of error we assume all gapfilling introduces the same amount of uncertainty (which isn’t the case), and our values overestimate uncertainty. In future research, incorporating estimates of actual gapfilling error will allow us to quantify the uncertainty introduced by gapfilling. This can lead to a systematic prioritization of improving data with the most influence on scores, as well as improving gapfilling approaches where no data acquisition is possible.

### Tracking gapfilling

To track and quantify gapfilling we generally mirrored the framework used to organize and calculate OHI data and scores. For each dataset (S1 Table), we created a “gapfilling dataset” that, for each observation (i.e., row of data) in the original dataset, described: 1) whether the value was gapfilled; 2) the method of gapfilling (if gapfilled); and, as a place-holder for future analyses, 3) the estimated error due to gapfilling. The percent contribution of gapfilled data to each OHI component score was calculated using the OHI models (S1 File). The contribution of gapfilled data to the final goal and index scores was presented using the same table and figure formats as OHI scores.

### Gapfilling datasets

For each dataset used in the OHI assessment, we describe the proportion of values that were gapfilled and the methods used to estimate missing data (S1 Table). Most of our gapfilling can be described by these general approaches:

1. Regional means: One of the most common methods of estimating missing data was to use the average values of regions sharing ecological, spatial, and/or political attributes relevant to the dataset being gapfilled. For several datasets (N = 12), we used geopolitical regions defined by the United Nations [31], which includes higher (N = 22) and lower (N = 7) resolution geopolitical regions. We preferentially used higher resolution data, but resorted to lower resolution data when there were too few regions with data within a geopolitical region to obtain a good estimate. In other cases, we used different regional classification systems. For coral reef condition we used averages (weighted by habitat area) of regions located in the same ocean basin (Fig S4 in [28]), and for seagrass condition we used seagrass regions as defined by Hemminga and Duarte [32]. For coral and seagrass condition and trend, we also gapfilled using values from neighboring regions.
2. Territorial disaggregation: Starting with the 2013 assessment, we began individually reporting territorial regions that had previously been grouped into classes based on administrative country and georegion characteristics (Table A in Supplement of [29]). For example, in the 2012 assessment we reported a single score for the Australian Tropical Territories comprised of Cocos Islands, Christmas Islands, and Norfolk Island; but after 2013, we reported individual scores for each of the three territories. For these datasets (N = 15), a single value was initially estimated for the entire group and then the individual territories were assigned the same value, or, an area weighted value (e.g., alien invasives).
3. Raster interpolation: For some datasets calculated using raster data (N = 2), we interpolated missing raster data and calculated the percentage of gapfilled cells for each region.
4. Correlates: Often the dataset being gapfilled was correlated with other datasets, and missing data were estimated using predictive models, such as linear regression (N = 4). For example, for the tourism and recreation goal, we gapfilled missing Travel and Tourism Competitiveness Index data [33] using a linear regression model with GDP and UN geopolitical region as predictor variables.
5. Zero-filled: For some datasets, we gapfilled missing data with zero values (N = 3: mariculture yield, natural products harvest, and targeted species harvest).
6. Temporal: For time series, we estimated missing years based on data from other years using regression models, or other techniques (e.g., a regression model was fit to available years of mangrove extent data to extrapolate current and/or reference years to estimate mangrove condition).
7. Taxonomic: When estimating missing data for organisms we used taxonomic averages. For example, for the mariculture sustainability data [34], we first averaged the most closely related taxa (e.g., different populations of the same species) and progressed, as necessary, to more distantly related taxa (e.g., species from the same genera, or order).
8. Combinations: For the FAO commodities harvest data [35], we used regression models to calculate the natural products goal. When tonnes of harvest was not reported for a commodity in a given year or region, we regressed export value in USD, year, and UN geopolitical region to predict these missing values. When both the harvest tonnes and harvest value were missing, we gapfilled these as zero values.

The best method of gapfilling missing values will vary according to the data being gapfilled, and is often limited by data availability. The best case scenario is when the dataset being gapfilled is closely related to the dataset used to estimate the missing data (e.g., harvest tonnes and harvest value come from the same data source). If these data aren’t available (which is usually the case), other datasets may still be useful for gapfilling missing values (e.g., the Social Progress Index may be correlated with the prevalence of waste water treatment, etc.). In many cases, the only available option for gapfilling missing data is to use the average of regions that are not missing data and share ecological, spatial, and/or political attributes relevant to the dataset being gapfilled. Whatever method is chosen, the data used to estimate missing values should be predictive of the dataset being gapfilled. For example, if the averages of UN geopolitical regions are used to estimate missing values, there should be statistically significant differences among the geopolitical regions (based on ANOVA, or other statistical test), if not, an overall mean of all the country data for all regions would probably be more appropriate for estimating missing data. The best approach, when possible, is to explore how well a variety of datasets predict missing values (e.g., harvest tonnes as a function of harvest value geopolitical region, year) by comparing the performance of models containing a combination of these variables (see section on “Estimating missing data using cross-validation”). Ultimately, the simplest model with the least predictive error should be chosen.

In some instances, it may be reasonable to assume that gapfilled data does not appreciably increase uncertainty in scores because it is estimated with little error. For example, we assumed that regions with no FAO inputs [36] for fertilizer consumption (nutrient pollution), pesticide consumption (organic pollution), or agricultural GDP were in fact zero values (N = 55 out of 220 countries had zero FAO input data). For the Convention on Biological Diversity signatories [37] data, we assigned territorial jurisdictions the same value as their administrative country and did not consider this gapfilling. Ultimately, these decisions are based on best expert judgement, and as such, may be modified as more information becomes available and methods evolve.

### Estimating missing data using cross-validation

For one of the ten goals, natural products, we were able to improve our method of gapfilling missing data. Specifically, we used leave-one-out cross-validation methods [38] to select among a series of candidate models that could be used to predict missing values in the data used to calculate the status and trend components of the goal. Cross-validation methods provide a rigorous method of assessing model performance and prevent the selection of overly complicated models. There are other robust methods for model selection, such as Akaike information criterion (AIC) and Bayesian information criterion (BIC), but an additional advantage of cross-validation is that it provides less biased estimates of error which can be used to estimate uncertainty and generate confidence intervals around index scores.

The FAO data used to calculate the natural products goal describes the tonnes and export value of each commodity produced in each country and year [35]. These data have several missing values for tonnes, but no missing values, in recent years, for export value. We compared how well several candidate regression models could predict the tonnes harvest of each commodity. All models included harvest value as a predictor, but varied in regard to whether year was included and the spatial scale of the analysis (country vs. UN geopolitical region vs. global). For each model we estimated error by removing one case (i.e., row of data) and fitting the model with the remaining data. We used the model estimates to predict the tons of harvest for the excluded case. We repeated this procedure for each row of data, and compared the observed and predicted values using Root Mean Square Error (RMSE, [39]).

### Influence of gapfilled data on OHI scores

We determined the relative contribution (i.e., weight) of each dataset to OHI scores, based on goal models (S1 File), to calculate the percent contribution of gapfilled data to the index scores (regional and global) as well as the component scores (status, trend, pressures, and resilience). Status scores have the largest contribution (88.3%) to index scores as a result of how the OHI is calculated. Consequently, any gapfilling in the datasets used to calculate status will have a large potential influence on uncertainty. We did not calculate percent gapfilling for the livelihoods and economies goal because the source data have been discontinued, in turn requiring a complete revision of how this goal is assessed. Future assessments of gapfilling will include this goal. Finally, we used regression models to determine whether gapfilling varied with region size and status (territorial region vs. administrative country). All analyses were conducted in R [40]. To calculate the standard deviation of EEZ area weighted goal scores (Table 2) we used the SDMTools package [41].

**Table 2:** Percent Contribution of Gapfilled Data to Global OHI Scores.

N = 220	Average %^a^		EEZ Weighted Average %^b^	
Goal (subgoal)	Average	SD	Average	SD
*Index (average of goals)*	25	17.8	19	16.8
*Artisanal Fishing Opportunities*	33	41.4	21	38.5
*Biodiversity*	22	19.2	18	17.8
* Habitats*	42	36.8	34	33.6
* Species*	2	2.4	1	2.3
*Coastal Protection*	28	21.0	18	21.1
*Carbon Storage*	29	20.3	22	20.6
*Clean Waters*	10	9.2	9	8.3
*Food Provisioning*	39	25.4	30	20.7
* Fisheries*	39	26.4	29	22.0
* Mariculture*	47	20.6	43	21.0
*Natural Products*	5	7.7	5	5.9
*Sense of Place*	1	2.2	1	2.2
* Iconic Species*	1	2.0	1	1.9
* Lasting Special Places*	1	2.7	1	2.6
*Tourism and Recreation*	40	43.2	30	42.4

^a^ average of the region index and goal scores: used when describing patterns of gapfilling among region scores.

^b^ average of the region index and goal scores after weighting the regions by their EEZ area (the standard presentation of OHI scores): used when describing patterns of gapfilling at the global scale because it better reflects the global coverage of gapfilling.

## Results

**Fig 1:**
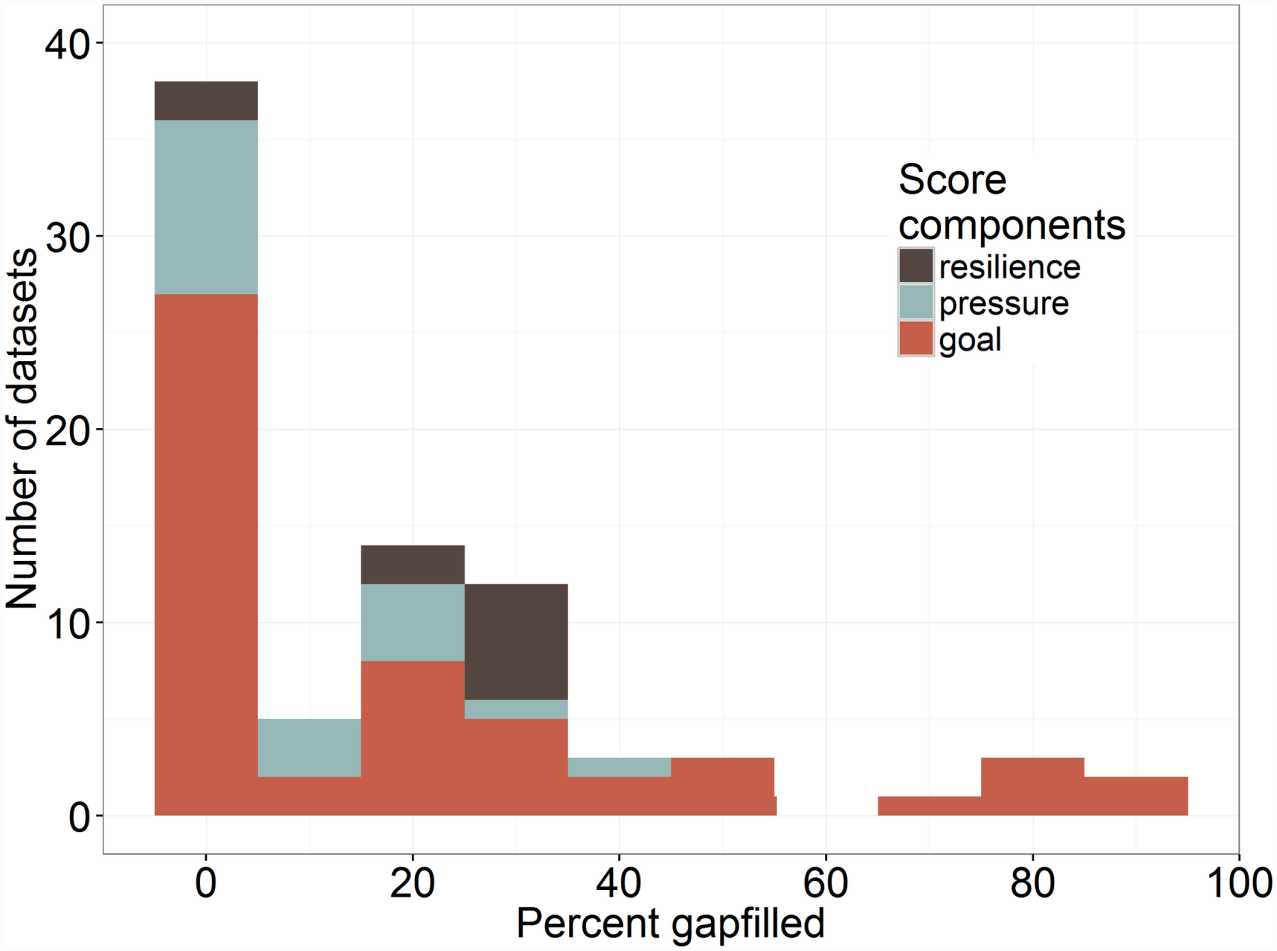
Percent Gapfilled Data. Histogram of percent gapfilled values in the datasets used to calculate OHI scores. Colors indicate which component of the OHI score (trend/status, pressure, resilience) the datasets were used to calculate.

For the 2015 OHI global assessment, about 58% of the datasets used to calculate OHI scores had some gapfilled data (Fig 1; S1 Table), and the percentage of gapfilled values ranged from 0–92%, with a mean of 18%. The datasets with the most gapfilling were used to assess the condition and trend of seagrass (92% and 82%, respectively) and coral habitats (79% and 53%, respectively), which are used to calculate coastal protection and carbon storage goals and the habitat subgoal of the biodiversity goal. In some cases large amounts of gapfilling may reflect the resolution of the original data. For example, the mariculture sustainability data [34] are specific to species and country; despite large amount of data, 69% of the data for species/country combinations had to be gapfilled. If the original data had less resolution (e.g., reporting country or species averages), the data would have required much less gapfilling but would not have resulted in less uncertainty in goal scores.

To determine the percent contribution of the gapfilled data to each OHI goal score (and subsequent regional and global index scores), we weighted the datasets based on the OHI models (S1 File). At the global scale, we found 18.5% of the overall global index score was based on gapfilled data, and the percent gapfilling for the individual goal/subgoal scores ranged from 1–43% (Table 2, EEZ weighted averages). The sense of place goal and the species and iconic species conservation subgoals had about 1% gapfilled data. The goals/subgoals with the highest average gapfilled data (although this can vary dramatically among regions, see below) were mariculture (subgoal of food provision), habitat (subgoal of biodiversity), tourism and recreation, and fisheries (subgoal of food provision).

**Fig 2:**
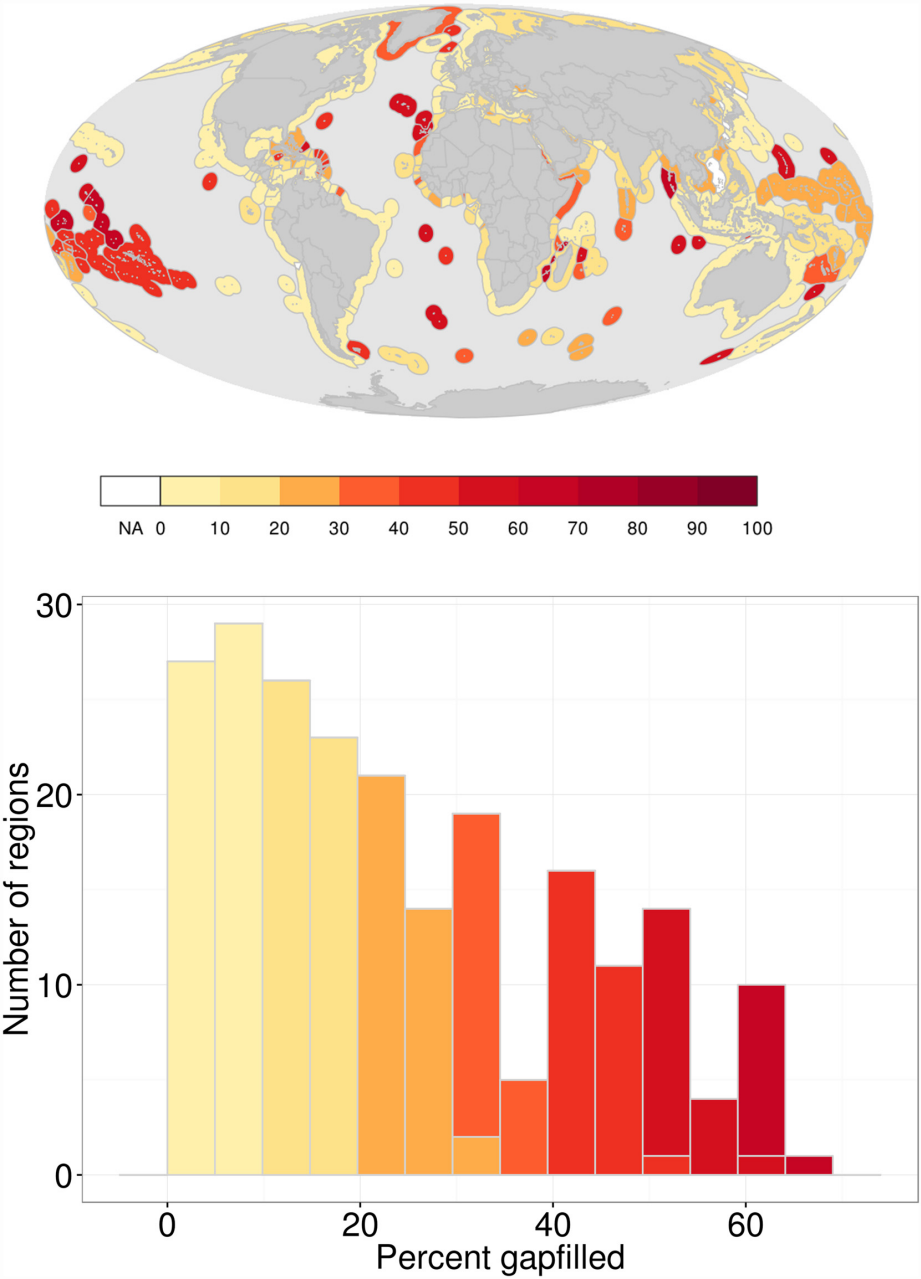
Percent Contribution of Gapfilled Data to Region Index Scores. Map describing the percent contribution of gapfilled data to index scores and histogram of index scores for 220 regions (subgoals in S1 Fig).

For the regions, the contribution of gapfilled data to their index scores ranged from 0 to 64% (Fig 2). For many regions, index scores were calculated using relatively little gapfilled data: 27 regions (of 220) had ≤ 5% gapfilled data, and 56 regions had ≤ 10%. The 10 regions with the most gapfilled data (≥ 60%) were small, often territorial, islands: Jersey and Guernsey (English Channel Islands, British Crown Dependencies); Oecussi Ambeno (a small coastal enclave in the western part of the Island of Timor); Andaman and Nicobar (Islands in Bay of Bengal, Indian territories); Glorioso Islands, Juan de Nova Island, Bassas da India and Ile Europa (French territories in the Indian Ocean around Madagascar); and, the Line and Phoenix Islands (Republic of Kiribati, Pacific Ocean). In general, the contribution of gapfilled data to index scores was fairly high for Islands in the South Pacific (Fig 2).

**Fig 3:**
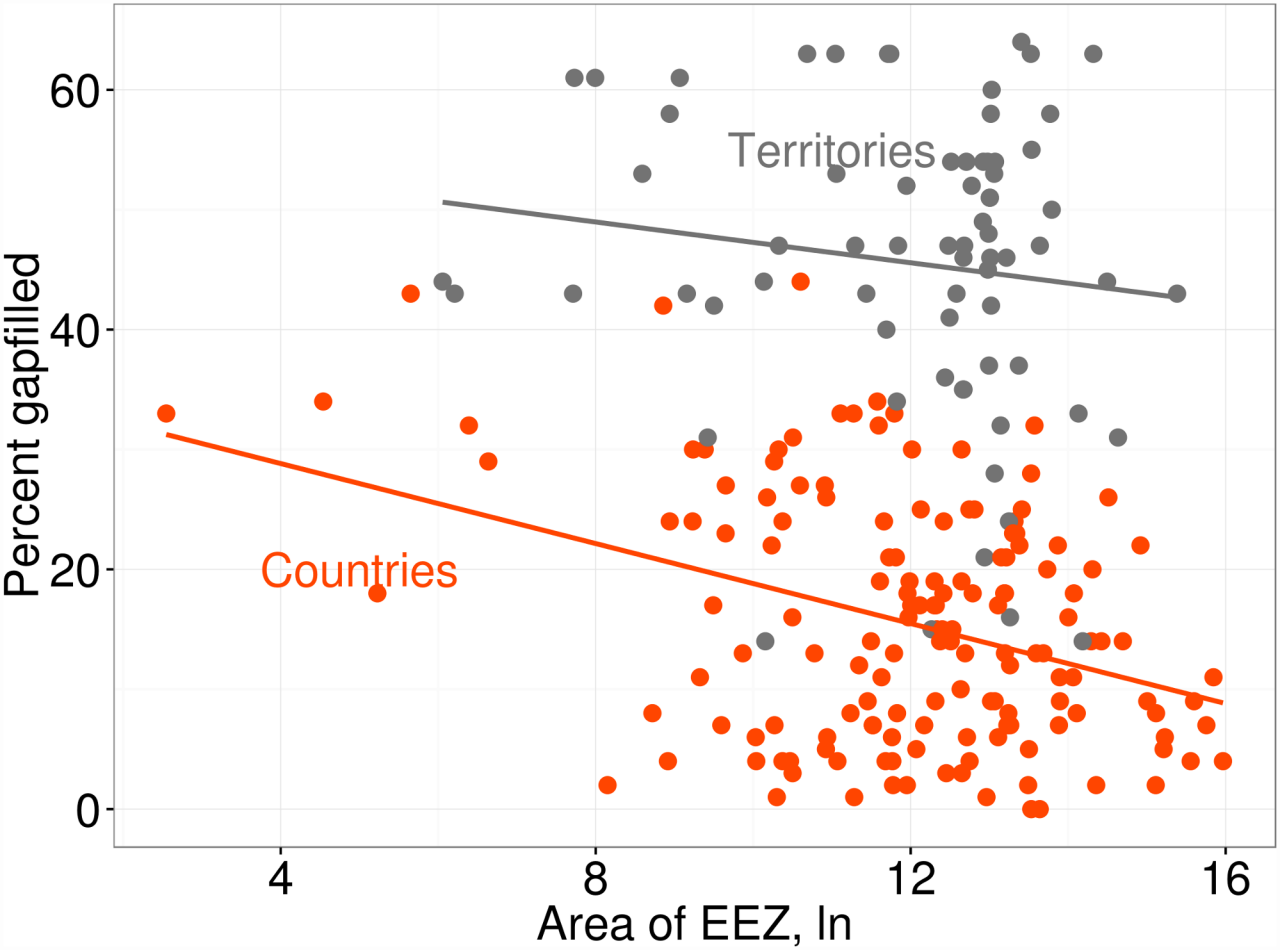
Predictors of the Contribution of Gapfilled Data to Region Index Scores. The contribution of gapfilled data to regional index scores was higher for smaller regions and territorial jurisdictions (see S2 file for model).

About 65% of the variation in gapfilling among region index scores was explained by region size and whether the region was a territorial jurisdiction of a country (Fig 3). Gapfilling tended to be higher for territories (P<0.001), and smaller regions (P<0.001; linear model, S2 File), with territorial status a much stronger predictor of gapfilling (R^2^ = 0.65 for model including both ln km^2^ and territorial status vs. R^2^ = 0.62 including only territorial status, S2 File).

**Fig 4:**
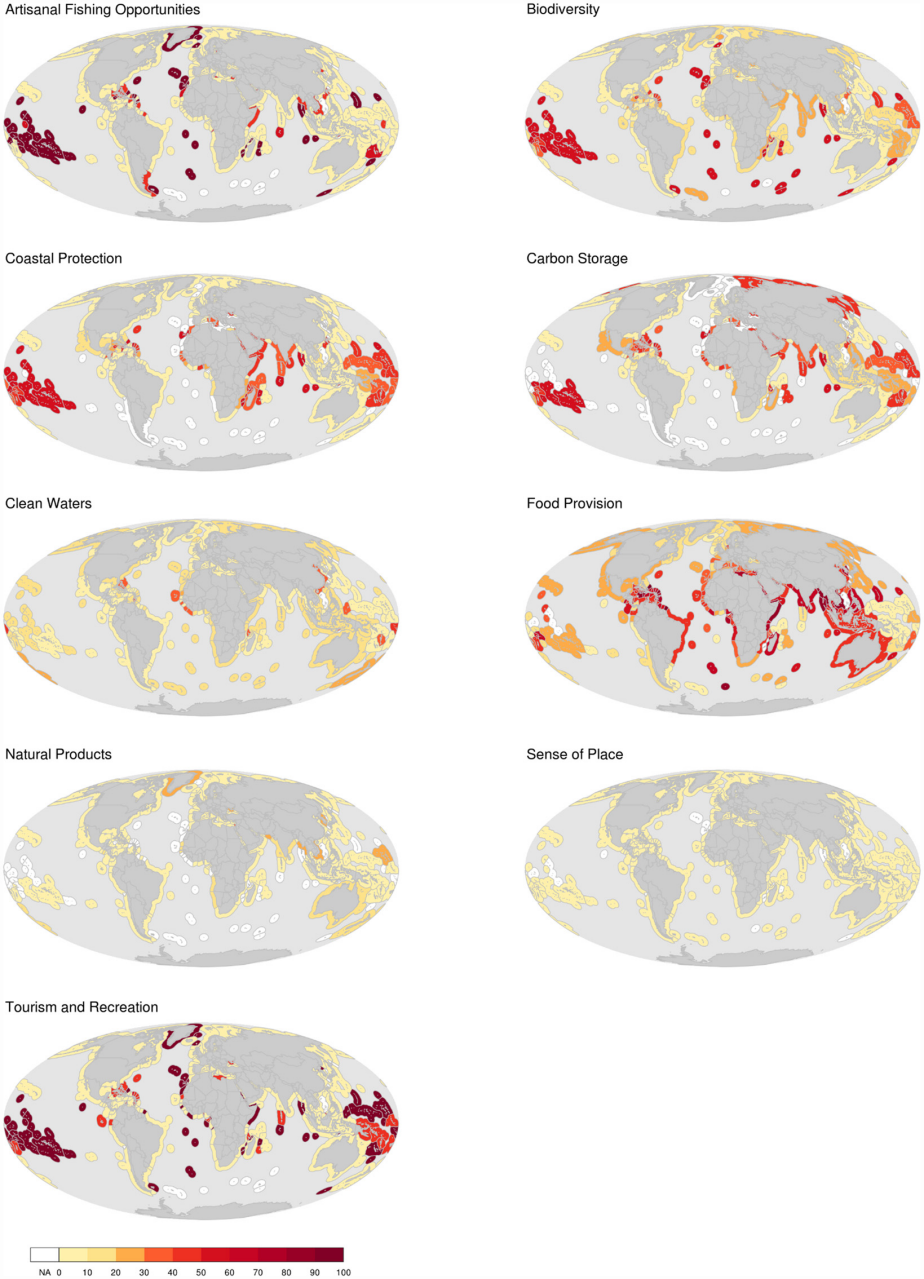
The Percent Contribution of Gapfilled Data to Goal Scores. Maps describing the contribution of gapfilling to 9 goal scores for all regions. Maps for subgoals used to calculate the food provision, sense of place, and biodiversity goals are available in S1 Fig.

For the region scores, the contribution of gapfilled data to goal scores ranged from 0–98% depending on the region and goal (Fig 4, S1 and S2 Figs, S2 Table). The mariculture subgoal tended to rely heavily on gapfilled data in most regions (gapfilling average and standard deviation = 47% ± 20.6, Table 2, average values). The fisheries subgoal was often calculated using large amounts of gapfilled data, even in regions with otherwise little gapfilling (gapfilling average and standard deviation = 39% ± 26.4, Table 2 average values). Gapfilling patterns for the coastal protection, carbon storage, and biodiversity (specifically, the habitat subgoal) were similar because these goals are calculated using the same habitat data.

Our application of cross-validation methods to estimate missing values in the data used to calculate the status and trend of the natural products goal demonstrates how estimates of gapfilling-derived error can be used to select the most parsimonious and accurate model among candidate models. By comparing the Root Mean Square Errors of the candidate approaches (S2 File), we found that models specific to each country had the least overall error, but many countries did not have enough data to apply this approach. In these cases, we estimated missing data with models specific to each UN geopolitical region, and if that was not possible, we resorted to using a global scale model (which assumes no spatial variation). For models at the UN geopolitical region and global scale, it was better to include year as a predictive variable (but not for models at the country scale). If the error of the global model had not been significantly higher, we would have adopted this simpler approach in all instances. However, cross-validation methods revealed that using the more complex models, whenever possible, greatly reduced error (RMSE: 3644 country model vs 6076 for global model), making the added complexity worthwhile.

## Discussion

Nearly half (42%) of all datasets used to calculate OHI scores had zero gapfilling, an additional 5% had <5% gapfilled values (Fig 1, S1 Table); and, overall, only 18.5% of the overall global index score was based on gapfilled data (Table 2). Given the challenges in finding good data with global coverage [42], these are notable and surprising results. Not surprisingly, the percent contribution of gapfilled data varied dramatically among regions and goals, with some regions/goals relying on large amounts of gapfilled data. The OHI scores for these regions have higher potential uncertainty.

Regions varied greatly in the contribution of gapfilled data to their overall index scores (Fig 2). Regions with larger amounts of gapfilled data tended to be smaller and/or territorial jurisdictions of countries (Fig 3, S2 File), highlighting the fact that most territories and smaller countries (often with smaller economies and other resources) tend to be monitored and assessed less frequently. Territorial jurisdictions had, on average, 30% more gapfilled data than similarly sized countries, reflecting the general tendency of administrative countries and/or reporting agencies to overlook these regions in regard to data collection and reporting. Although territorial OHI scores have low reliability due to gapfilling, these regions should still be reported because their scores include local information when possible, and thus are more accurate than assigning them the scores of their administrative countries (which is what effectively would happen if they were grouped with their administrative country) or not evaluating them at all (which, literally, leaves them off the map). Furthermore, quantifying the amount of gapfilling for these regions makes explicit the potential uncertainty in their scores, thus incentivizing future efforts to reduce data gaps for these regions.

The proportion of gapfilled data varied dramatically among goals and subgoals (Fig 4; S1 and S2 Figs; S2 Table). Many regions had large amounts of gapfilling for tourism and recreation, artisanal fishing opportunities, food provisioning, and the habitat related goals (carbon storage, coastal protection, biodiversity). For the tourism and recreation and artisanal fishing opportunities goals, the large amount of required gapfilling was due to the exclusion of smaller countries and territorial jurisdictions by the agencies providing the data used to calculate these goals.

One of the goals with the most gapfilling was food provisioning (Fig 4), primarily due to the fisheries subgoal (S1 and S2 Figs). Mariculture actually had the largest percentage of gapfilled data of any goal/subgoal (S1 and S2 Figs), but its contribution to food provisioning was small compared to fisheries for most regions. Fisheries data were gapfilled when catch was not reported to the species level, which is common in global fisheries data [43]. For these taxa, stock status scores (*B/B*_*msy*_) cannot be calculated and were thus gapfilled using the median scores of stocks from the same region and year (Supplementary materials in [28]). The implication of large amounts of missing values for fisheries datasets is difficult to interpret because it may reflect poor fisheries management (because effective harvest targets cannot easily be set for a species when using higher taxonomic catch information) or simply poor data reporting to the FAO. The former situation implies that data-limited stocks are more likely to be overharvested, while the latter situation simply implies less certainty about stock status (in theory, species could be doing better or worse than the taxonomic average). A further complication is that, at least in some cases, stocks can be well managed at the genus or family level in some regions, particularly in tropical regions with high species diversity [44], which is where fisheries gapfilling appeared highest.

Several of the habitat datasets were heavily gapfilled (particularly seagrass and coral reef condition and trend data). These datasets are used to calculate multiple OHI goals (carbon storage, coastal protection, and biodiversity), which means the impact of missing habitat data is greater than for other datasets. Furthermore, these datasets are known to have several data quality issues that result in a low degree of confidence in our estimates of missing data [45]. The lack of global, high resolution, repeated measures of habitat extent and quality for the vast majority of the world’s oceans remains one of the most vexing challenges for nearly every ocean condition assessment [42], and certainly for the Ocean Health Index. This information suggests that investing in better habitat data collection would significantly reduce the uncertainty of the global OHI assessment, as well as other environmental assessments.

Gapfilling is an important aspect of indicator development for several reasons. Beyond, allowing us to use available data, it can reduce bias, and when done transparently, it provides a measure of the reliability of scores and highlights how data collection can be improved. Gapfilling can actually reduce bias in results [46,47]. For example, if we avoided gapfilling datasets used to calculate a goal in which most countries tend to score poorly, the overall index score for those regions with missing data would most likely be inflated due to this omission. As such, it is often better to gapfill than to not, especially because it is usually possible to make reasonable estimates of missing data. Future research could incorporate sensitivity analyses to better understand how gapfilling (vs. no gapfilling) influences region and global scores. More importantly, the error introduced by gapfilling can be explicitly quantified and reported, thus providing a measure of the reliability of the scores for different regions and components. The transparent documentation of gapfilling can also motivate better data collection, research, and/or reporting. Showing the effect of poor quality information on the assessment of issues that people care about, such as OHI scores, often provides a powerful incentive to improve data collection efforts.

There are several avenues for future work. A major limitation of our assessment is that we do not directly estimate uncertainty introduced by gapfilling. Instead, we estimate the *potential* contribution of gapfilled data to the uncertainty of each index score, based on which values were gapfilled in a dataset (e.g., which regions), and the dataset’s relative weight in the models used to derive scores. This approach provides a first approximation of the uncertainty introduced by gapfilling and helps identify which geographic regions and goals require greater data collection efforts in the future. Nonetheless, it is important to keep in mind that scores with equal contributions of gapfilled data probably do not have equal uncertainty. Ultimately, we will generate confidence intervals around OHI scores using cross-validation estimates of error due to gapfilling missing data. Improved assessments of uncertainty will better inform how gapfilling influences scores and potentially identify situations when gapfilling is not an appropriate option.

Although there are many sources of uncertainty for composite models [22–25], we only evaluate one source of uncertainty. A full treatment and estimate of all sources of uncertainty is extraordinarily difficult as many sources are simply unknowable or difficult to measure. Furthermore, we can only provide an account of the gapfilling that we performed ourselves; in many cases, data will have a prior history of gapfilling that is not documented. As more datasets begin to track gapfilling more explicitly in an accessible and standardized format, such ‘hidden gapfilling’ will be less of an issue. Thus, tracking and reporting gapfilling is a useful first step in the move towards more comprehensive evaluations of all sources of uncertainty.

## Conclusions

The standard for nearly all scientific inquiry is to present results alongside measures of uncertainty, and this standard should equally apply to composite indicators. Estimates of uncertainty are critical for evaluating conclusions and informing how data can be responsibly used. The results of scientific inquiry are often based on standard statistical models (ANOVA, regression models, etc.) that have well-defined methods of estimating and describing uncertainty (estimates of error, confidence intervals, p-values, etc.). For composite indicator models, however, dealing with uncertainty is more complicated, and consequently, rarely implemented. Despite the challenges, it is possible to quantify uncertainty in composite indicators [14,15,20,21,26,27]. One of our goals here was to inform gapfilling efforts during future indicator construction. The following describes what we have learned from our efforts to track, quantify, and describe gapfilling for the global OHI assessment. This information is especially useful for regional OHI assessments [48], which use the same data framework as the global assessment, but can be used for any indicator development.

1. *Create a robust system early for tracking gapfilling*. We found the best approach for tracking gapfilling was to mirror the general framework used to organize, and calculate the index data and scores.
2. *Choose the best gapfilling method using analytical tests (when possible)*. Often multiple approaches are available to gapfill missing data. It is worth taking the time to compare these options because they will vary in their predictive ability. In these cases, cross-validation is a good method of comparing models because it can be used to select the most parsimonious model that minimizes error, so as to avoid selection of unnecessarily complicated models. In contrast to AIC and BIC model selection methods (which are also good options for choosing among candidate models), cross-validation methods also provide a more accurate estimate of error than those provided by the outputs of regression or ANOVA methods. These error estimates can be used to generate confidence intervals around scores.
3. *There is always more to do*. The documentation of uncertainty should be considered an iterative process that will improve over time.

The information presented here is useful at many levels. The quality of global OHI scores for different regions and goals can now be more transparently assessed. A better understanding of the shortcomings of the underlying data also helps focus efforts to improve global OHI models and data sources. This information is particularly valuable to researchers conducting regional OHI assessments. OHI is a conceptual and analytical framework that can be tailored and applied to any spatial scale, and one of the primary objectives of the OHI Project (http://ohi-science.org/) is to provide tools and data to facilitate these assessments [48]. Many of these efforts rely, at least partially, on global data, and this information, in combination with some understanding of the degree of error associated, can be used to prioritize data collection efforts that will have the highest gains in terms of reduction of uncertainty.

## Supporting Information

S1 Fig Maps of Subgoal Gapfilling. Percent contribution of gapfilled data to subgoal scores of food provision, sense of place, and biodiversity goals.
(TIF)

S2 Fig Heatmap of Regional Gapfilling. Percent contribution of gapfilled data to index and goal scores for each region.
(TIFF)

S1 File Supporting Methods. Additional information on how datasets were gapfilled, and detailed description of how scores and score components (status, trend, pressure, resilience) were calculated.
(DOCX)

S2 File Supporting Results. Results from cross-validation for the harvest tonnes data and model exploring relationship between gapfilling and regional characteristics.
(DOCX)

S1 Table Methods of Gapfilling Datasets. Describes how each dataset used in the OHI models was gapfilled and the proportion of gapfilled values.
(DOCX)

S2 Table Percent Gapfilling of Regional Scores Data. Dataset (csv file) describing the percent contribution of gapfilled data to index and goal scores for each region.
(CSV)
