# Supplementary figures and images for: Mapping Uncertainty Due to Missing Data in the Global Ocean Health Index
Source: PLoS One. 2016 Aug 2;11(8):e0160377. doi: 10.1371/journal.pone.0160377 (PMC4970671; doi:10.1371/journal.pone.0160377)

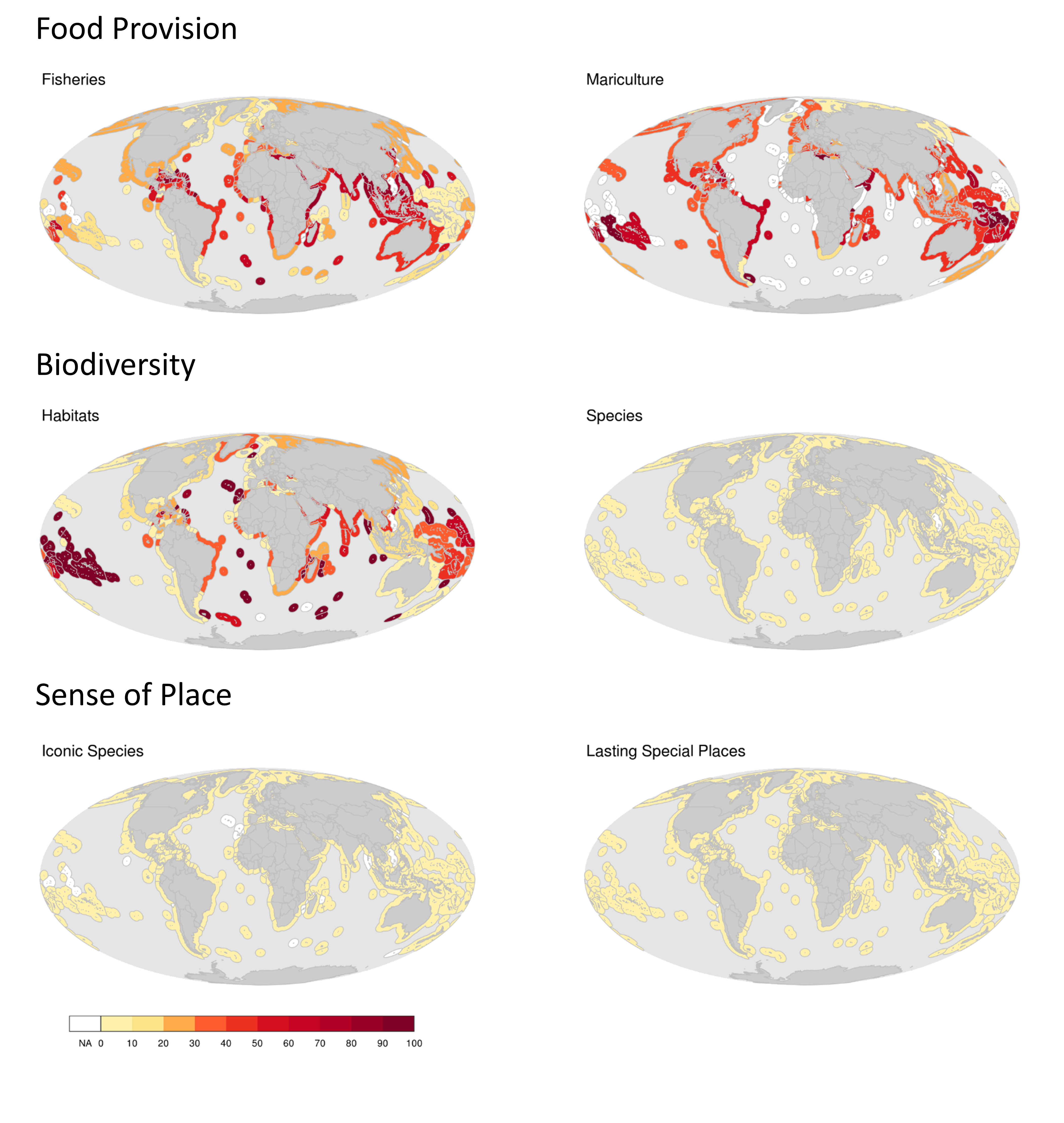

Supplement: S1 Fig — Percent contribution of gapfilled data to subgoal scores of food provision, sense of place, and biodiversity goals. (TIF) [file pone.0160377.s001.tif]

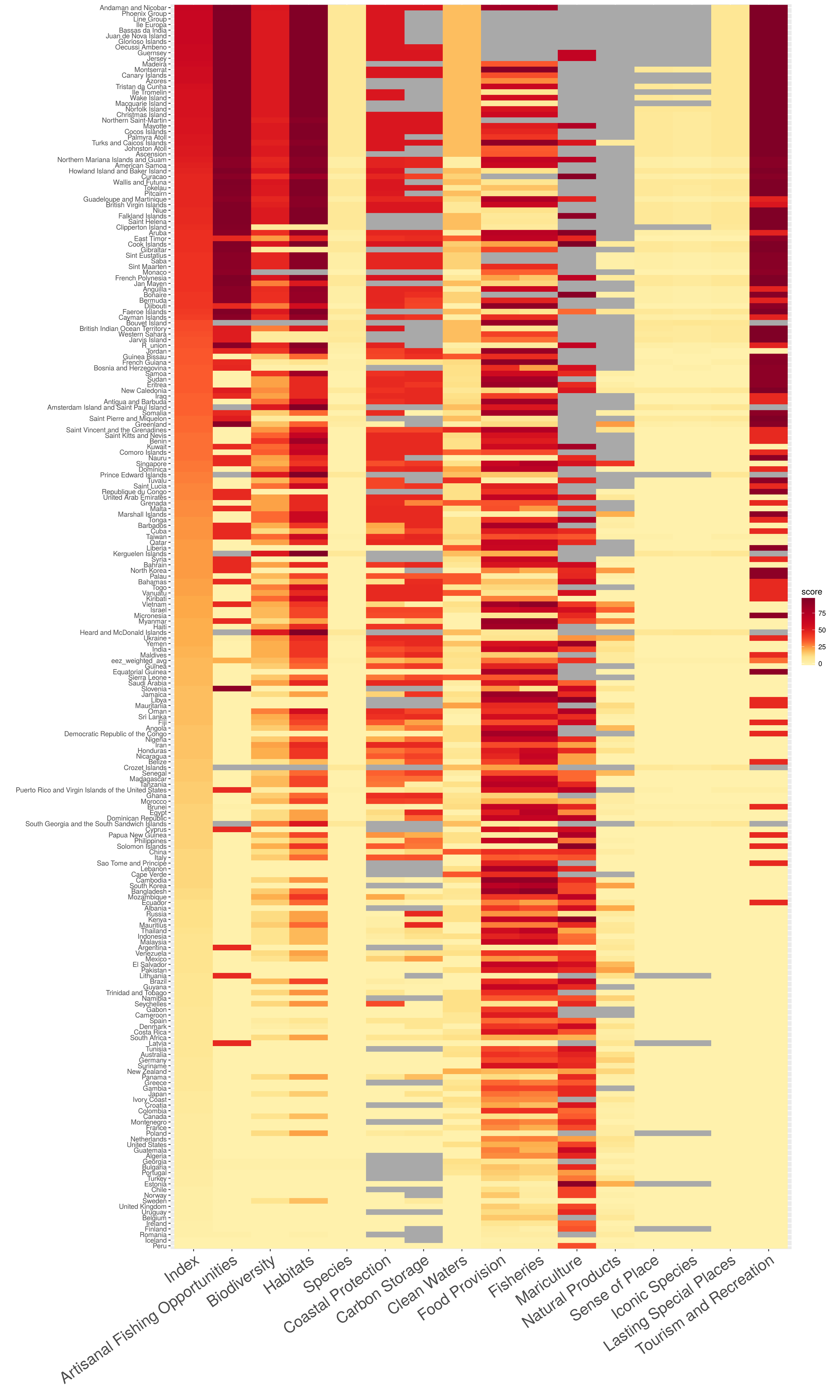

Supplement: S2 Fig — Percent contribution of gapfilled data to index and goal scores for each region. (TIFF) [file pone.0160377.s002.tiff]
