# Supplementary material for: Mapping Uncertainty Due to Missing Data in the Global Ocean Health Index
Source: PLoS One. 2016 Aug 2;11(8):e0160377. doi: 10.1371/journal.pone.0160377 (PMC4970671; doi:10.1371/journal.pone.0160377)
Supplement: S1 File — Additional information on how datasets were gapfilled, and detailed description of how scores and score components (status, trend, pressure, resilience) were calculated. (DOCX) [file pone.0160377.s003.docx]

S1 File. Supporting Methods

We tracked which values were gapfilled in each of the datasets used to calculate the Ocean Health Index (S1 Table). We then determined the relative contribution of each dataset, based on the OHI models (Halpern et al., 2015, 2012), to the goal and index scores. The following provides an overview of these methods.

## 1. Proportion of gapfilling for a region’s Index score

A region’s overall index score is the average of 10 goal scores that pertain to how people interact with and benefit from marine systems. Consequently, the proportion of gapfilled data in each region’s index score was calculated by averaging the proportion of gapfilled data for the 10 goals.

## 2. Proportion of gapfilling for goal scores

Each goal’s score is an average of its current status and projected future status. The current status compares the current condition of each goal to a defined reference point (Section 3). The projected future status is an estimate of a goal’s condition after five years based on expected changes in the current status imputed from recent trends in status (Section 3) and current pressures and resilience (Section 4) components acting on each goal.

The proportion of gapfilling for each goal score,$I_{i\_gf}$, was calculated as:

$$I_{i\_gf}= \frac{x_{i\_gf}+ \hat{x}_{i,F\_gf}}{2}$$

given the proportion of gapfilled data used to calculate current status, $x_{i\_gf}$, and likely near-term future status:

$$\hat{x}_{i,F\_gf}=[1+ \beta T_{i\_gf}+(1-\beta)(r_{i\_gf}- p_{i\_gf})x_{i\_gf}]$$

Which was the proportion of gapfilled data used to calculate the trend ($T_{i\_gf}$), pressure ($p_{i\_gf}$), and resilience ($r_{i\_gf}$) components of each goal; and a fixed Beta (β) value of 0.67 which weights the contribution of the trend component twice as high as the resilience and pressure components in determining the likely trajectory of the goal status into the future.

## 3. Proportion of gapfilling for status and trend scores

The following describes how we calculated the proportion of gapfilled data in the status and trend components of the scores. Status is a measure of the current condition of a goal relative to a reference point. The trend describes the average yearly change in status, based on a linear regression of the five most recent years of status data, and then projected five years into the future (i.e., the slope estimate is multiplied by 5). The percentage of gapfilled data in trend scores, unless otherwise noted, was calculated by averaging the proportion of gapfilled data of the 5 most recent years of status data.

### 3.1 Food Provision

For the food provision goal, the proportion of gapfilled data was calculated by averaging the fisheries and mariculture subgoal values weighted by the tonnes of wild caught fish vs. mariculture yield.

#### 3.1.1 Fisheries

Calculating fisheries status involves several steps, but the core model calculates the geometric mean of the status scores (*SS*) for stocks landed within each region/year, weighted by each stock’s proportion of the total catch (averaged across all years within a region):


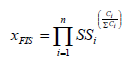


Where *i* is an individual taxon and *n* is the total number of taxa in the reported catch for that region throughout the time series, and *C* was calculated as the taxon average catch for each reporting region in each year across the catch time-series starting at the first non-null record.

The stock status scores are based on *B/B_msy_* estimates of the population biomass relative to the biomass that can deliver maximum sustainable yield for each landed stock. The *B/B_msy_* values were obtained using Martell and Froese’s (2013) data-limited model (referred to as catch-MSY) to estimate *B/B_msy_* values using catch data provided by Sea Around Us (Pauly and Zeller, 2015). For the OHI model, the *B/B_msy_* values were then converted to stock status scores that range between 0-1, and penalize over- (*B/B_msy_* < 1) and under-harvesting (*B/B_msy_* > 1).

Unfortunately, *B/B_msy_* values cannot be calculated using the catch-MSY method for all taxa with catch data, and the *B/B_msy_* values for these taxa are estimated using gapfilled data. Most frequently this occurs because taxa are not reported to the species level, which is required for the catch-MSY model. Ideally, all catch data would reported to the species level, but in reality catch is often reported at higher taxonomic levels, such as genus, family, and, in the worst cases, “Marine fishes not identified”. The catch data can also be inadequate for other reasons (e.g., too small a sample size, failure to converge, etc.). We gapfilled these data using the median *B/B_msy_* values of the stocks from the same region and year. A penalty is applied to gapfilled *B/B_msy_* values when the stock is not identified to species, as this is considered an indicator of a poorly managed stock.

The Fisheries gapfilling status for each region and year was calculated as:

$$x_{FIS\_gf}= \sum_{1}^{k} \left( {B/B_{msy}}_{k\_gf}\times\frac{C_{k}}{C} \right)$$

with each stock’s (*k*) gapfilled *B/B_msy_* value (*B/B_msy k_gf_*, 0 or 1 value) multiplied by the stock’s average catch (*C_k_*) divided by the total average catch within each region (*C*), and then summed for all taxa within the region. Catch was calculated as the taxon average for each of the reporting regions in each year across the catch time-series since the first record of catch. The average catch over time was selected as it reflects the mean potential contribution of each species to total food provision, independent of stochastic fluctuations and possible recent declines.

#### 3.1.2 Mariculture

The mariculture status subgoal is based on the sustainably-harvested yield (*Y_c_*) within each region:

$$Y_{c}=\sum_{1}^{k} Y_{k}S_{k}$$

Where *Y_k_* is the 4-year moving window average harvest tonnes for all *k* mariculture species that are currently, or at one time cultured within a country and *S*_k_ is the sustainability score for each *k* mariculture species. To calculate the status score, the Y_c_ values generated for each region are divided by the coastal population, and divided by the reference Yc value, defined as the 95^th^ quantile of *Y_c_* values among the countries.

Given the equal contributions of the yield and sustainability variables, we calculated the total proportion of gapfilling (*x_mar_gf_*) for each region as:

$$x_{MAR\_gf}= \frac{\sum_{1}^{k} {(S}_{k\_gf}+Y_{k\_gf})/2}{k}$$

where, each species (*k*) gapfilled sustainability score (*S_k_gf_*, 0 or 1 value) and harvest yield score (*Y_k_gf_*, 0 or 1 value) were averaged. These values were summed for all the species within a region and divided by the total number of species.

### 3.2 Artisanal Fishing Opportunities

For artisanal opportunities, the status score is reduced when there is a high economic need and is increased when there is good access to artisanal fishing opportunities (Mora et al., 2009):

$$x_{AO}=1-[need\times\left( 1-access \right)]$$

where, *need* is estimated as (1 – *PPPpcGDP*), with *PPPpcGDP* calculated as the log-transformed, rescaled purchasing power parity adjusted by per capita GDP (World Bank, 2013); and, *access* is based on data from Mora et al. (2009) Given the equal contributions of *need* and *access*, the gapfilling score was calculated for each region, as:

$$x_{AO\_gf}= \frac{{access}_{gf}+ {need}_{gf}}{2}$$

where, *access_gf_* and *need_gf_*, have a 1 value if gapfilled, or otherwise 0.

### 3.3 Natural Products

The natural products status and trend are calculated using harvest data for commodities that are assigned to one of 6 categories of non-food natural products (United Nations, FAO commodities data): coral, sponges, ornamentals, fish oil, shells, seaweeds. The status of each product is calculated as:

$$x_{p}=H_{p} \times S_{p}$$

where *H_p_* is the harvest tonnes relative to the maximum harvest tonnes within a region with a 35% buffer (i.e., max harvest tonnes * 0.65). And, *S_p_* is the sustainability score for the product, calculated as:

$$S_{p}= \frac{E+R}{2}$$

Where, *E* is exposure (harvest amount relative to harvest environment) and *R* is risk.

To calculate overall status, the average of the product status (*x_p_*) scores is calculated, weighted by relative dollar value (dollar value provides a more meaningful comparison than weight).

The harvest tonnes and exposure data were gapfilled, while the harvest value and risk data were not. To calculate the proportion of each natural product calculated using gapfilled data for each country and reporting year:

$$H_{p\_gf}=\frac{\sum_{i=1}^{N} h_{gf}h_{t}}{\sum_{i=1}^{N} h_{t}}$$

Where, N is the number of commodities harvested in each region and year. If the commodity is gapfilled, *h_gf_* has a 1 value, and otherwise 0. And, *h_t_* is the harvest tonnes for each commodity. To calculate gapfilling for each product:

$$x_{p\_gf}= H_{p\_gf}\times0.5 + E_{gf}\times0.25 + R_{gf}\times0.25$$

Where *E_gf_* and *R_gf_* represent gapfilling for the exposure and risk terms, and had a value of 1 if gapfilled, or otherwise 0.

To calculate overall gapfilling for status and trend, the gapfilled status scores for each product ($x_{p\_gf}$) were averaged, weighted by relative dollar value (dollar value provides a more meaningful comparison among products than tonnes):

$$x_{NP\_gf}=\frac{\sum_{i=1}^{N} w_{p}{\times x}_{p\_gf}}{N}$$

Where *N* is the number of products that have ever been harvested in a region and *w_p_* is the proportional peak dollar value of each product.

### 3.4 Carbon Storage

Carbon storage was calculated by averaging the condition of the carbon storing marine habitats (mangrove, saltmarsh, and seagrass) present in a region, weighted by each habitat’s area and carbon storage capacity. Condition and trend data were gapfilled and area was not. Condition and trend were calculated using different datasets, and consequently, have different patterns of gapfilling. The proportion of gapfilled data used in status scores, was calculated as:

$$x_{CS\_gf}=\frac{\sum_{h=1}^{N} {A_{h}\times w}_{h}\times g_{h\_gf}}{\sum_{h=1}^{N} A_{h}\times w_{h}}$$

Where, *N* is the number of habitats (*h*) within a region, *A_h_* is the area extent (km^2^), and *w_h_* is carbon storage capacity (Table S1-1). The *g_h_gf_* term indicates the proportion of data gapfilled, with a value of 1 if both condition and area were gapfilled, 0.5 if one of the variables was gapfilled, and 0 if no gapfilling.

The proportion of gapfilled data used in trend scores, was calculated as above, but gapfilling for trend data was used in place of condition.

**Table S1-1: Carbon storage capacity of marine habitats.**

| **Habitat** | **Carbon capacity** |
| --- | --- |
| Mangrove | 139 |
| Saltmarsh | 210 |
| Seagrass | 83 |

### 3.5 Coastal Protection

Coastal protection is calculated by averaging the condition of the marine habitats present in a region that protect shorelines (coral, mangrove, saltmarsh, seagrass, and shoreline seaice), weighted by the habitat’s area and rank protection score. For these habitats (except seaice, which had no gapfilling), condition and trend data were often gapfilled, whereas, area of the habitat was not. Condition and trend were calculated using different datasets, and consequently, could have different patterns of gapfilling. The proportion of gapfilled data used in status scores, was calculated as:

$$x_{CP\_gf}=\frac{\sum_{h=1}^{N} {A_{h}\times w}_{h}\times g_{h\_gf}}{\sum_{h=1}^{N} A_{h}\times w_{h}}$$

Where, *N* is the number of habitats (*h*) within a region, *A_h_* is the area extent (km^2^), and *w_h_* is the rank protection (Table S1-2). The *g_h_gf_* term indicates the proportion of data gapfilled, with a value of 1 if both condition and area were gapfilled, 0.5 if one of the variables was gapfilled, and 0 if no gapfilling.

The proportion of gapfilled data used in trend scores, was calculated as above, but gapfilling for trend data was used in place of condition.

**Table S1-2: Shoreline protection rank scores for marine habitats.**

| **Habitat** | **Rank shoreline protection** |
| --- | --- |
| Coral | 4 |
| Mangrove | 4 |
| Saltmarsh | 3 |
| Seagrass | 1 |
| Sea ice (shoreline) | 4 |

### 3.6 Tourism & Recreation

Tourism and recreation status is calculated as:

$$x_{TR}=E_{p}+S$$

where, *E_p_* is the proportion of employment in tourism and recreation and *S* is the sustainability score. The proportion of gapfilling was calculated as:

$$x_{TR\_gf}=\frac{E_{p\_gf}+S_{gf}}{2}$$

Where *E_p_gf_* and *S_gf_* represent gapfilling for the employment and sustainability terms, and had a value of 1 if gapfilled, or otherwise 0.

### 3.7 Sense of Place

The proportion of gapfilling for the sense of place goal was the average of the lasting special places and iconic species subgoals.

#### 3.7.1 Iconic Species

There was no gapfilling for iconic species status or trend calculations.

#### 3.7.2 Lasting Special Places

There was no gapfilling for lasting special places status or trend calculations.

### 3.8 Clean Waters

Clean waters status and trend are based on 4 pollution pressures: pathogens, fertilizers, chemicals, and trash (coastal human population is used as a proxy to estimate trash *trend*, because global trash data is not available across years). To calculate clean water status, the geometric mean of these pressures (scaled from 0-1) is calculated, after they are subtracted from one (to convert pressure into status).

The proportion of gapfilled data in the clean waters status was calculated as as:

$$x_{CW\_gf}=\frac{\sum_{i=1}^{N} p_{gf}}{N}$$

Where, *N* is the number of pressures within a region (some regions do not have all 4 pressures), and *p_gf_* has a value of 1 if the pressure is gapfilled, and otherwise, 0.

### 3.9 Biodiversity

The proportion of gapfilling for the biodiversity goal was the average of the species and habitats subgoals.

#### 3.9.1 Species

There was no gapfilling for species status or trend calculations.

#### 3.9.2 Habitats

The habitat goal averages the condition of the marine habitats, within a region, including: coral, mangrove, saltmarsh, seagrass, seaice (edge and shoreline), and soft bottom. For these habitats, condition and trend data were often gapfilled (seaice had no gapfilling) and area was not. Condition and trend were calculated using different datasets, and consequently, could have different patterns of gapfilling. The proportion of gapfilled data used in status scores, was calculated as:

$$x_{CP\_gf}=\frac{\sum_{h=1}^{N} c_{h\_gf}}{N}$$

Where, *N* is the number of habitats (*h*) within a region, and *c_h_gf_* indicates whether habitat condition was gapfilled, with a value of 1 if gapfilled, and otherwise 0.

The proportion of gapfilled data used in trend scores, was calculated as above, but *t_h_gf_* replaced the *c_h_gf_* variable.

### 3.10 Livelihoods and Economies

At this time, the livelihoods and economies goal and subgoals are not included because the models are being revised due to changes in data.

## 4. Proportion of gapfilling for pressure and resilience scores

Each goal may have several pressure and resilience variables that influence future status. For example, the pressures acting on seagrass habitat are: chemical and nutrient pollution, invasive species, intertidal habitat destruction, and sea surface temperature anomalies, ocean acidification, and sea level rise (see pressure matrix: <https://github.com/OHI-Science/ohi-global/blob/draft/eez2015/conf/pressures_matrix.csv>). The resilience variables that buffer some of the pressures on seagrass habitat are: regulations to prevent invasive species, establishment of marine protected areas, water quality regulations, and controls on mariculture and tourism (see resilience matrix: https://github.com/OHI-Science/ohi-global/blob/draft/eez2015/conf/resilience_matrix.csv). The pressure variables are multiplied by a value of 0-3 to reflect their relative impact on each goal. The resilience variables are weighted to reflect the quality of the data. The pressure and resilience variables are aggregated to calculate the pressure and resilience components for each goal and region (see Eqs. S7-S13 in Supplementary Information, Halpern et al., 2012).

To determine the proportion of gapfilling for the pressure and resilience components for each goal and region, we tracked whether the values in each pressure or resilience dataset were gapfilled (Table S1). We then used the OHI models, pressure/resilience matrices, and weights to determine the proportion of the pressure and resilience scores that were calculated using gapfilled data.

## References

Halpern, B.S., Longo, C., Hardy, D., McLeod, K.L., Samhouri, J.F., Katona, S.K., Kleisner, K., Lester, S.E., O/’Leary, J., Ranelletti, M., Rosenberg, A.A., Scarborough, C., Selig, E.R., Best, B.D., Brumbaugh, D.R., Chapin, F.S., Crowder, L.B., Daly, K.L., Doney, S.C., Elfes, C., Fogarty, M.J., Gaines, S.D., Jacobsen, K.I., Karrer, L.B., Leslie, H.M., Neeley, E., Pauly, D., Polasky, S., Ris, B., St Martin, K., Stone, G.S., Sumaila, U.R., Zeller, D., 2012. An index to assess the health and benefits of the global ocean. Nature 488, 615–620. doi:10.1038/nature11397

Halpern, B.S., Longo, C., Lowndes, J.S.S., Best, B.D., Frazier, M., Katona, S.K., Kleisner, K.M., Rosenberg, A.A., Scarborough, C., Selig, E.R., 2015. Patterns and emerging trends in global ocean health. PLoS ONE 10, e0117863. doi:10.1371/journal.pone.0117863

Martell, S., Froese, R., 2013. A simple method for estimating MSY from catch and resilience. Fish Fish. 14, 504–514. doi:10.1111/j.1467-2979.2012.00485.x

Mora, C., Myers, R.A., Coll, M., Libralato, S., Pitcher, T.J., Sumaila, R.U., Zeller, D., Watson, R., Gaston, K.J., Worm, B., 2009. Management effectiveness of the world’s marine fisheries. PLoS Biol. 7, e1000131. doi:10.1371/journal.pbio.1000131

Pauly, D., Zeller, D. (Eds.), 2015. Sea Around Us Concepts, Design and Data.

United Nations, n.d. FAO Fisheries & Aquaculture - Fishery Statistical Collections - Fishery Commodities and Trade [WWW Document]. URL http://www.fao.org/fishery/statistics/global-commodities-production/en (accessed 4.13.16).

World Bank, 2013. PPPpcGDP.
