# Supplementary material for: Mapping Uncertainty Due to Missing Data in the Global Ocean Health Index
Source: PLoS One. 2016 Aug 2;11(8):e0160377. doi: 10.1371/journal.pone.0160377 (PMC4970671; doi:10.1371/journal.pone.0160377)
Supplement: S2 File — Results from cross-validation for the harvest tonnes data and model exploring relationship between gapfilling and regional characteristics. (DOCX) [file pone.0160377.s004.docx]

S2 File. Supporting Results

**Cross-validation results**. Comparison of the performance of several candidate models to predict the harvest tonnes of marine commodities (used to calculate natural product goal). Models varied in regard to their spatial scale (within a country, georegion, global) and whether year was included as a predictor variable (all models included the harvest value in USD as a predictor). Error (RMSE) was estimated for all models by comparing observed vs. predicted data based on leave-one-out cross validation (CV RMSE) and standard methods (RMSE (no CV)).

| **Model** | **CV RMSE** | **RMSE (no CV)** |
| --- | --- | --- |
| Country scale | 3644 | 2790 |
| Country scale + year | 5053 | 2513 |
| UN georegion scale | 6082 | 5390 |
| UN georegion scale + year | 5141 | 5129 |
| Global scale | 6519 | 6220 |
| Global scale + year | 6076 | 6066 |

**Gapfilling and region size and territorial status.** Linear regression candidate models describing how the percent contribution of gapfilled data to region Index scores varies according to region size (ln km^2^) and status as a territorial jurisdiction (F_2, 217_=200.4, R^2^ = 0.65, P<0.001).

| **Model** | **Coefficient** | **Estimate** | **Std. Error** | **t value** | **P-value** | **R^2^** | **AIC** |
| --- | --- | --- | --- | --- | --- | --- | --- |
| Model 1 (best performing) | Intercept | 32.809 | 4.1412 | 7.923 | <0.001 | 0.65 | 1668 |
|  | ln (km^2^) | -1.444 | 0.3385 | -4.264 | <0.001 |  |  |
|  | territory | 30.097 | 1.5346 | 19.612 | <0.001 |  |  |
| Model 2 | Intercept | 35.498 | 4.8318 | 7.347 | <0.001 | 0.65 | 1669 |
|  | ln (km^2^) | -1.668 | 0.3974 | -4.198 | <0.001 |  |  |
|  | territory | 20.279 | 9.2272 | 2.198 | 0.029 |  |  |
|  | ln (km^2^) x territory | 0.8180 | 0.7580 | 1.079 | 0.282 |  |  |
| Model 3 | Intercept | 15.540 | 0.899 | 17.29 | <0.001 | 0.62 | 1684 |
|  | territory | 30.017 | 1.594 | 18.83 | <0.001 |  |  |
| Model 4 | Intercept | 41.410 | 6.841 | 6.053 | <0.001 | 0.02 | 1890 |
|  | ln (km^2^) | -1.362 | 0.5623 | -2.422 | 0.0162 |  |  |
