# Supplementary material for: Mapping Uncertainty Due to Missing Data in the Global Ocean Health Index
Source: PLoS One. 2016 Aug 2;11(8):e0160377. doi: 10.1371/journal.pone.0160377 (PMC4970671; doi:10.1371/journal.pone.0160377)
Supplement: S1 Table — Describes how each dataset used in the OHI models was gapfilled and the proportion of gapfilled values. (DOCX) [file pone.0160377.s005.docx]

S1 Table. Gapfilling Datasets

Description of gapfilling methods for datasets used in the 2015 OHI assessment to calculate component scores for: status, trend, pressure and resilience. Refer to SOM of Halpern et al. 2015 for complete description of datasets.

| **Dataset** | **Brief Description** | **Goal Component** | **Reference** | **Gapfilling method** | **Proportion gapfilled (most recent year of data)** |  |
| --- | --- | --- | --- | --- | --- | --- |
| Fisheries catch data | Global fisheries catch statistics in yield per species | FIS  (Status, Trend)  FP (weights) | Pauly and Zeller 2015 | None | 0 |  |
| Stock exploitation status (*B/B_msy_*) | Exploitation status of fished stocks | FIS (Status, Trend) | United Nations 2013, Martell and Froese 2013 | Median of stocks in same region/year | 0.48 |  |
| Mariculture Sustainability Index (MSI): taxa/country specific mariculture sustainability | Mariculture Sustainability Index (MSI): fishmeal use, waste treatment, and seed and larvae origin indicators | MAR (Status, Trend) | Trujillo 2008 | MSI is country and taxa-specific. If a country has mariculture yield for a taxa but no corresponding MSI, the taxa specific average from other countries is used. If a country farms a species that was not assessed at all by the MSI but a species within the same genus was assessed, a global average for the genus was used. Finally, if these scores were not available for the categories above, we used the global average for broad taxonomic grouping (e.g., crustaceans, algae,  bivalves, etc.). | 0.69 |  |
| Mariculture yield | Tonnes production of finfish and invertebrates | MAR (Status, Trend)  FP (weights) | United Nations | Missing values gapfilled with zeros. | 0.18 |  |
| Artisanal fishing management effectiveness and opportunity | Artisanal fishing opportunities | AO (Status, Trend) Resilience | Mora et al. 2009, Fig. S4 | Average scores of UN geopolitical regions; territories within an administrative country/georegion (S2 Table) were estimated as a single group and then assigned the same value | 0.33 |  |
| Economic need | Gross Domestic Product, per capita, adjusted by Purchasing Power Parity (PPPpcGDP) | AO (Status, Trend) | World Bank 2013 | Average scores of UN geopolitical regions | 0.40 |  |
| Natural products harvest | Export (in tons) of coral, ornamental fish, fish oil, sponges, shells, and seaweeds and plants | NP (Status, Trend) | United Nations | Estimated missing tonnes for each commodity using regression models with export value in USD as a predictor variable. If a country had adequate data, the model was based on the most recent 10 years of commodity data within the country (tons_rgn_ ~ USD_rgn_). Otherwise, the model was based on the commodity data within a UN geopolitical regions and reporting year (tons_georgn_ ~ USD_georgn_ + year). In some cases, there was not enough data at the georegion scale, and the model was based on the global data. When both the value and harvest datasets had missing values we gapfilled these as zeros. | 0.02 |  |
| Natural products value | Export revenue (US dollars) | NP (Status, Trend) | United Nations | None | 0 |  |
| Natural products risk | Assigned risk for Natural Products.  Ornamentals: blast and cyanide fishing practices | NP (Status, Trend) | Burke et al. 2011,  CITES 2013 | None | 0 |  |
| Natural products exposure | coral, sponges, ornamentals, shells, seaweeds: calculated using harvest relative to area of coral and rocky reef habitat.  Fish oil: Fish status | NP (Status, Trend) | See habitat data | Coral, sponges, ornamentals, shells, seaweeds: gapfilled when a region produced a natural product but there was no corresponding habitat data, suggesting gaps in habitat data. These were gapfilled with the mean exposure in other regions. | 0.22 |  |
| Habitat: Rocky reef | Global rocky reef habitat extent | NP exposure component (Status, Trend) | Halpern et al. 2008 | Not accounted for in this analysis, but is estimated: “Rocky Reef intertidal data do not exist globally so rocky reef habitat was assumed to exist in all cells within 1km of shore globally.” (Halpern et al., 2012), SOM) | 0 |  |
| Habitat: Coral reefs | Global coral habitat extent, condition, and trend | CP, CS, HAB, NP_exposure_ (Status, Trend) | Burke et al. 2011, Bruno and Selig, 2007, Schutte et al. 2010, Halpern et al., 2008 | Extent: None  Condition: Average of adjacent EEZs and/or EEZs within the same ocean basin weighted by habitat area (Figure S4 from Halpern et al. 2012(Halpern et al., 2012)); territories within an administrative country/georegion (S2 Table) were estimated as a single group and then assigned the same value  Trend: Territories within an administrative country/georegion (S2 Table) were estimated as a single group and then assigned the same value | Extent: 0  Condition: 0.79  Trend: 0.53 |  |
| Habitat: Mangroves | Global mangrove habitat from remote sensing and assessments | CP, CS, HAB (Status, Trend) | Hamilton and Casey 2016 (extent and trend); United Nations 2007 (condition) | Extent: None  Condition: Regression models used to extend the years of data when reference and/or current time periods were not available to calculate condition; territories within an administrative country/georegion (S2 Table) were estimated as a single group and then assigned the same value  Trend: Three regions reported together and assigned same value | Extent: 0  Condition: 0.25  Trend: 0.06 |  |
| Habitat: Salt marsh | Global salt marsh habitat extent | CP, CS, HAB (Status, Trend) | Bridgham et al. 2006, Dahl 2011, EEA, New Zealand and Ministry for the Environment 2007, JNCC 2004 | Extent: None. However, there are countries with known salt marsh habitat have no area in these data (Halpern et al. 2012, SOM)  Condition & trend: Territories within an administrative country/georegion (S2 Table) were estimated as a single group and then assigned the same value | Extent: 0  Condition: 0.24  Trend: 0.24 |  |
| Habitat: Sea ice | Sea ice change in extent, both edge and shoreline metrics | CP, HAB (Status, Trend) | Cavalieri et al. 1996 | None | Extent: 0  Condition: 0  Trend: 0 |  |
| Habitat: Seagrass | Global seagrass habitat extent and change in condition | CP, CS, HAB (Status, Trend) | UNEP-WCMC and FT 2005, Waycott et al. 2009, Short et al. 2011 | Extent: None  Condition: Average of adjacent EEZs and/or EEZs within the same seagrass regions as defined by Hemminga and Duarte (reference, Table S24 in Halpern et al. 2012, SOM) weighted by habitat area; territories within an administrative country/georegion (S2 Table) were estimated as a single group and then assigned the same value.  Trend: Territories within an administrative country/georegion (S2 Table) were estimated as a single group and then assigned the same value | Extent: 0  Condition: 0.92  Trend: 0.82 |  |
| Habitat: Soft-bottom subtidal | Global soft-bottom subtidal habitat extent, and modeled status for change in condition | HAB (Status, Trend) Pressure (inverse) | Halpern et al. 2008, Pauly and Zeller 2015 | Extent: None  Condition & trend: Territories within an administrative country/georegion (S2 Table) were estimated as a single group and then assigned the same value | Extent: 0  Condition: 0.24  Trend: 0.24 |  |
| Travel and Tourism Competitiveness Index (TTCI) | Sustainability of the travel and tourism industry | TR (Status, Trend) | World Economic Forum, 2015 | Linear regression model with GDP and UN geopolitical regions as predictors. In a few cases, GDP was estimated using UN geopolitical regions data. | 0.50 |  |
| Travel and Tourism Direct Contribution to Employment | Employment directly linked to travel and tourism sectors (hotels, transportation, services) | TR (Status, Trend) | WTTC | UN geopolitical regions averages | 0.34 |  |
| Iconic species list | WWF Priority and Flagship Species Lists | ICO (Status, Trend) | Halpern et al. 2012,  WWF | None | 0 |  |
| Marine species extinction risk status | IUCN threat category; sub-population status for iconic species | ICO, SPP (Status)  Resilience | IUCN 2015,  Kaschner et al. 2015 | None | 0 |  |
| Marine species population trend | IUCN population trend category; sub-population status for iconic species | ICO, SPP (Trend) | IUCN 2015,  Kaschner et al. 2015 | None | 0 |  |
| Marine species ranges | IUCN and Aquamaps range maps | ICO, SPP (Status, Trend)  Resilience | IUCN 2015,  Kaschner et al. 2015 | None | 0 |  |
| Nutrient pollution: land based nitrogen | Modeled N input from fertilizer use | CW (Status, Trend) Pressure | Halpern et al. 2015, United Nations | Missing values filled in by regression between fertilizer and pesticides when possible, and when not possible with agricultural GDP as a proxy (Halpern et al. 2008). | 0.06 |  |
| Chemical pollution: land-based inorganic | Modeled pollution from urban runoff from impervious surfaces | CW (Status, Trend) Pressure | Halpern et al. 2015, Homer et al. 2004 | None | 0 |  |
| Chemical pollution: land-based organic | Modeled pollution from pesticides | CW (Status, Trend) Pressure | Halpern et al. 2015, 2008, United Nations | Missing values filled in by regression between fertilizer and pesticides when possible, and when not possible with agricultural GDP as a proxy (Halpern et al. 2008) | 0.39 |  |
| Chemical pollution: ocean-based | Modeled pollution from shipping and ports | CW (Status, Trend) Pressure | Halpern et al. 2015, 2008 | None | 0 |  |
| Pathogen pollution: Coastal human population density | Human population density within 25 mi from shore | CW (Status, Trend) Pressure | CIESIN 2005a | None | 0 |  |
| Pathogen pollution: Sanitation | Proportion of population without access to improved sanitation facilities | CW (Status, Trend)  Pressure | WHO-UNICEF | UN geopolitical regions averages | 0.3 |  |
| Trash pollution | Marine ocean plastic | CW (Status)  Pressure | Eriksen et al. 2014 | None | 0 |  |
| Coastal human population | Human population within 25 mi from shore | CW (Trend) MAR (Status, Trend) | CIESIN 2005b | None | 0 |  |
| Marine protected areas and terrestrial protected areas | Relative area of MPAs within EEZ waters or within 3 nm of shore, and relative area of designated protected areas (CP) within 1 km of shore | LSP (Status, Trend) Resilience | UNEP-WCMC 2015 | None | 0 |  |
| Alien species | Number of alien species per marine ecoregion | Pressure | Molnar et al. 2008 | Original data for MEOW ecoregions, and then downscaled to regions.  Territorial regions within an administrative country/georegion group (S2 Table) were estimated as one region, and this value was weighted by the relative proportion of each territory’s eez area to assign values to each territorial region | 0.27 |  |
| Artisanal fishing: high bycatch | Presence of artisanal blast and poison fishing practices | Pressure | Burke et al. 2011 | Territories within an administrative country/georegion group (S2 Table) were estimated as one region and then assigned the same value | 0.24 |  |
| Artisanal fishing: low bycatch | Modeled low bycatch artisanal fishing using non-destructive practices based on FAO fisheries statistics | Pressure | Halpern et al. 2008 | None  (Note: these data were gapfilled previous to OHI that we do not take into account, Halpern et al. 2008) | 0 |  |
| Commercial fishing: high bycatch | Modeled demersal and pelagic high bycatch fishing pressure | Pressure | Halpern et al. 2008, Behrenfeld and Falkowski 1997 | UN geopolitical regions gapfilling for a few regions (Bulgaria, Romania, Georgia, Ukraine, Jordan). Bosnia gapfilled with Croatia data. Bouvet Island given a zero value given surrounding area had so little fishing. | 0.03 |  |
| Commercial fishing: low bycatch | Modeled demersal and pelagic low bycatch fishing pressure | Pressure | Halpern et al. 2008, Behrenfeld and Falkowski 1997 | UN geopolitical regions gapfilling for a few regions (Bulgaria, Romania, Georgia, Ukraine, Jordan). Bosnia gapfilled with Croatia data. Bouvet Island given a zero value given surrounding area had so little fishing. | 0.03 |  |
| Convention on Biological Diversity (CBD) signatories | List of 192 countries who signed CBD | Resilience | Convention on Biological Diversity | territories assigned value of administrative country (not considered gapfilling) | 0 |  |
| Convention on Biological Diversity (CBD) survey | Answers to questions relating to alien species, habitat, mariculture, tourism, and water | Resilience  Alien species  Habitat  Mariculture  Tourism  Water | Secretariat of the Convention on Biological Diversity 2005 | CBD data were gapfilled using UN geopolitical regions averages, weighted by areas of countries with data; territories within an administrative country/georegion group (S???) were estimated as one region and then assigned the same value. | 0.32 |  |
| Convention on International Trade in Endangered Species of Wild Fauna and Flora (CITES) signatories | List of countries who signed CITES | Resilience | CITES 2013 | None | 0 |  |
| Mariculture Sustainability Index (MSI): mariculture sustainability and mariculture regulations | Mariculture Sustainability Index (MSI): “traceability” and “code of conduct” measures | Resilience | Trujillo 2008 | Territories within an administrative country/georegion (S2 Table) were estimated as a single group and then assigned the same value | 0.21 |  |
| Fisheries management effectiveness | Management effectiveness of the world’s marine fisheries | Resilience  Used in calculation of combined resilience layers:  Fishing v1 Fishing v1 eez Fishing v2 eez Fishing v3 Fishing v3 eez | Mora et al. 2009 | Average scores of UN geopolitical regions; territories within an administrative country/georegion (S2 Table) were estimated as a single group and then assigned the same value | 0.33 |  |
| Genetic escapes | Mariculture Sustainability Index (MSI): native or introduced indicator | Pressure | Trujillo 2008 | MSI is country and taxa-specific. If a country has mariculture yield for a taxa but no corresponding MSI, the taxa specific average from other countries is used. If a country farms a species that was not assessed at all by the MSI but a species within the same genus was assessed, a global average for the genus was used. Finally, if these scores were not available for the categories above, we used the global average for broad taxonomic grouping (e.g., crustaceans, algae,  bivalves, etc.). | 0.37 |  |
| Habitat destruction: intertidal | Population density within 10 km of the shore | Pressure | CIESIN 2005a | None | 0 |  |
| Habitat destruction: subtidal hard bottom | Presence of blast and poison artisanal fishing practices | Pressure | Burke et al. 2011 | Territories within an administrative country/georegion group (S2 Table) were estimated as one region and then assigned the same value | 0.24 |  |
| Habitat destruction: subtidal soft bottom | Presence of trawling practices in soft bottom habitats | Pressure | Halpern et al. 2008 | None | 0 |  |
| Ocean acidification | Change in aragonite saturation state (ASS) levels | Pressure | Feely et al. 2009 | Spatial interpolation of raster data. Average gapfilling relatively low, but some regions were heavily gapfilled. | 0.19 |  |
| Sea level rise | Net change in sea level during the time series | Pressure | AVISO 2015 | Spatial interpolation of raster data. Most regions had no gapfilled raster cells. Republique du Congo had the most gapfilling with 29%. | 0.005 |  |
| Sea surface temperature (SST) anomalies | Sea surface temperature anomalies | Pressure | Selig et al. 2010 | None | 0 |  |
| Targeted harvest | Catch statistics for cetaceans and marine turtles | Pressure | United Nations 2013 | Gapfilled with zero scores for regions without FAO reporting. | 0.18 |  |
| UV radiation | Anomalies in intensity of ultraviolet (UV) radiation | Pressure | Thaminnen and Arola 2013 | None | 0 |  |
| Worldwide Governance Indicators (WGI) | Accountability, Political Stability and Absence of Violence, Government Effectiveness, Regulatory Quality, Rule of Law, and Control of Corruption | Pressure (1 - WGI), Resilience | Kaufmann et al. 2010 | UN geopolitical regions averages | 0.24 |  |

AVISO, 2015. Satellite altimetry data. http://www.aviso.altimetry.fr/en/data/products/ocean-indicators-products/mean-sea-level (accessed 1.12.15).

Behrenfeld, M.J., Falkowski, P.G., 1997. Photosynthetic rates derived from satellite-based chlorophyll concentration. Limnol. Oceanogr. 42, 1–20.

Bridgham, S.D., Megonigal, J.P., Keller, J.K., Bliss, N.B., Trettin, C., 2006. The carbon balance of North American wetlands. Wetlands 26, 889–916. doi:10.1672/0277-5212(2006)26[889:TCBONA]2.0.CO;2

Bruno, J.F., Selig, E.R., 2007. Regional decline of coral cover in the Indo-Pacific: timing, extent, and subregional comparisons. PLoS ONE 2, e711. doi:10.1371/journal.pone.0000711

Burke, L., Reytar, K., Spadling, M., Perry, A., Cooper, E., Kushner, B., Selig, E., Starkhouse, B., Teleki, K., Waite, R., Wilkinson, C., Young, T., 2011. Reefs at risk - revisited. World Resources Institute, Washington, D.C.

Cavalieri, D.J., Parkinson, C.L., Gloersen, P., Zwally, H.J., 1996. Sea ice concentrations from Nimbus-7 SMMR and DMSP SSM/I-SSMIS passive microwave data, version 1. NASA National Snow and Ice Data Center Distributed Active Archive Center, Boulder, Colorado USA.

CIESIN, Center for International Earth Science Information Network, Columbia University, CIAT, Centro Internacional de Agricultura Tropical, 2005a. Gridded population of the world, version 3 (GPWv3): population density grid. Palisades, NY: NASA Socioeconomic Data and Applications Center (SEDAC).

CIESIN, Center for International Earth Science Information Network, Columbia University, United Nations Food and Agriculture Programme - FAO, Centro Internacional de Agricultura Tropical - CIAT, 2005b. Gridded population of the world, version 3 (GPWv3): population count grid. Palisades, NY: NASA Socioeconomic Data and Applications Center (SEDAC).

CITES, 2013. Member countries. https://cites.org/eng/disc/parties/index.php

Convention on Biological Diversity. List of parties. https://www.cbd.int/convention/ (accessed 4.13.16).

Dahl, T.E., 2011. Status and trends of wetlands in the conterminous United States 2004 to 2009. US Department of the Interior, US Fish and Wildlife Service, Fisheries and Habitat Conservation.

EEA (Eionet, European Environment Agency). EU Habitats Directive Article 17 Reporting, 2001-2006. http://bd.eionet.europa.eu/activities/Reporting/Article_17/reference_portal

Eriksen, M., Lebreton, L.C.M., Carson, H.S., Thiel, M., Moore, C.J., Borerro, J.C., Galgani, F., Ryan, P.G., Reisser, J., 2014. Plastic pollution in the world’s oceans: more than 5 trillion plastic pieces weighing over 250,000 tons afloat at sea. PLoS ONE 9, e111913. doi:10.1371/journal.pone.0111913

Feely, R., Doney, S., Cooley, S., 2009. Ocean acidification: present conditions and future changes in a high-CO2 world. Oceanography 22, 36–47. doi:10.5670/oceanog.2009.95

Halpern, B.S., Frazier, M., Potapenko, J., Casey, K.S., Koenig, K., Longo, C., Lowndes, J.S., Rockwood, R.C., Selig, E.R., Selkoe, K.A., Walbridge, S., 2015. Spatial and temporal changes in cumulative human impacts on the world’s ocean. Nat. Commun. 6, 7615. doi:10.1038/ncomms8615

Halpern, B.S., Longo, C., Hardy, D., McLeod, K.L., Samhouri, J.F., Katona, S.K., Kleisner, K., Lester, S.E., O/’Leary, J., Ranelletti, M., Rosenberg, A.A., Scarborough, C., Selig, E.R., Best, B.D., Brumbaugh, D.R., Chapin, F.S., Crowder, L.B., Daly, K.L., Doney, S.C., Elfes, C., Fogarty, M.J., Gaines, S.D., Jacobsen, K.I., Karrer, L.B., Leslie, H.M., Neeley, E., Pauly, D., Polasky, S., Ris, B., St Martin, K., Stone, G.S., Sumaila, U.R., Zeller, D., 2012. An index to assess the health and benefits of the global ocean. Nature 488, 615–620. doi:10.1038/nature11397

Halpern, B.S., Walbridge, S., Selkoe, K.A., Kappel, C.V., Micheli, F., D’Agrosa, C., Bruno, J.F., Casey, K.S., Ebert, C., Fox, H.E., Fujita, R., Heinemann, D., Lenihan, H.S., Madin, E.M.P., Perry, M.T., Selig, E.R., Spalding, M., Steneck, R., Watson, R., 2008. A global map of human impact on marine ecosystems. Science 319, 948–952. doi:10.1126/science.1149345

Hamilton, S., Casey, D., 2016. Creation of a high spatiotemporal resolution global database of continuous mangrove forest cover for the 21st Century (CGMFC-21). Glob. Ecol. Biogeogr. doi:10.1111/geb.12449

Homer, C., Huang, C., Yang, L., Wylie, B., Coan, M., 2004. Development of a 2001 national land-cover database for the United States. Photogramm. Eng. Remote Sens. 70, 829–840.

IUCN, 2015. Red List of Threatened Species. http://www.iucnredlist.org/

JNCC (Joint Nature Conservation Committee), 2004. Common standards monitoring guidance for saltmarsh habitats.

Kaschner, K., Kesner-Reyes, K., Garilao, C., Rius-Barile, J., Rees, T., Froese, R., 2015. AquaMaps: Predicted range maps for aquatic species.

Kaufmann, D., Kraay, A., Mastruzzi, M., 2010. The Worldwide Governance Indicators: methodology and analytical issues (SSRN Scholarly Paper No. ID 1682130). Social Science Research Network, Rochester, NY.

Martell, S., Froese, R., 2013. A simple method for estimating MSY from catch and resilience. Fish Fish. 14, 504–514. doi:10.1111/j.1467-2979.2012.00485.x

Molnar, J.L., Gamboa, R.L., Revenga, C., Spalding, M.D., 2008. Assessing the global threat of invasive species to marine biodiversity. Front. Ecol. Environ. 6, 485–492. doi:10.1890/070064

Mora, C., Myers, R.A., Coll, M., Libralato, S., Pitcher, T.J., Sumaila, R.U., Zeller, D., Watson, R., Gaston, K.J., Worm, B., 2009. Management effectiveness of the world’s marine fisheries. PLoS Biol. 7, e1000131. doi:10.1371/journal.pbio.1000131

New Zealand, Ministry for the Environment, 2007. Environment New Zealand 2007. Ministry for the Environment, Wellington, N.Z.

Pauly, D., Zeller, D. (Eds.), 2015. Sea Around Us Concepts, Design and Data.

Schutte, V., Selig, E., Bruno, J., 2010. Regional spatio-temporal trends in Caribbean coral reef benthic communities. Mar. Ecol. Prog. Ser. 402, 115–122. doi:10.3354/meps08438

Secretariat of the Convention on Biological Diversity, 2005. Handbook of the Convention on Biological Diversity (No. 3rd edition). Montreal, Canada.

Selig, E.R., Casey, K.S., Bruno, J.F., 2010. New insights into global patterns of ocean temperature anomalies: implications for coral reef health and management. Glob. Ecol. Biogeogr. 19, 397–411. doi:10.1111/j.1466-8238.2009.00522.x

Short, F., Coles, R., Fortes, M., Koch, E., 2011. SeagrassNet. http://www.seagrassnet.org/ (accessed 5.9.16).

Thaminnen, J., Arola, A., 2013. Aura OMI global surface UVB data product-OMUVBd (Version 003). NASAGSFC Greenbelt MD USA NASA Goddard Earth Sci. Data Inf. Serv. Cent. GES DISC.

Trujillo, P., 2008. Using a mariculture sustainability index to rank countries’ performance, in: Alder, J., Pauly, D. (Eds.), Fisheries Centre Research Reports. Fisheries Centre Research Reports, University of British Columbia, Vancouver, Canada.

UNEP-WCMC, 2015. World Database on Protected Areas User Manual.

UNEP-WCMC, FT, S., 2005. Global Distribution of Seagrasses (version 3). Third update to the data layer used in Green and Short (2003), superseding version 2. UNEP World Conservation Monitoring Centre, Cambridge, UK.

United Nations, 2013. FAO Fisheries & Aquaculture - Fishery Statistical Collections - Global Capture Production. http://www.fao.org/fishery/statistics/global-capture-production/en

United Nations, 2007. The world’s mangroves 1980-2005. FAO Forestry Paper 153.

United Nations. FAO Fisheries & Aquaculture - Fishery Statistical Collections - Global Aquaculture Production. http://www.fao.org/fishery/statistics/global-aquaculture-production/en (accessed 4.13.16a).

United Nations. FAO Fisheries & Aquaculture - Fishery Statistical Collections - Fishery Commodities and Trade. http://www.fao.org/fishery/statistics/global-commodities-production/en (accessed 4.13.16b).

United Nations. FAO Statistics Division - Inputs. http://faostat3.fao.org/browse/R/*/E (accessed 4.13.16c).

Waycott, M., Duarte, C.M., Carruthers, T.J.B., Orth, R.J., Dennison, W.C., Olyarnik, S., Calladine, A., Fourqurean, J.W., Heck, K.L., Hughes, A.R., Kendrick, G.A., Kenworthy, W.J., Short, F.T., Williams, S.L., 2009. Accelerating loss of seagrasses across the globe threatens coastal ecosystems. Proc. Natl. Acad. Sci. 106, 12377–12381. doi:10.1073/pnas.0905620106

WHO-UNICEF. Joint Monitoring Programme (JMP) for Water Supply and Sanitation. http://www.wssinfo.org/

World Bank, 2013. PPPpcGDP.

World Economic Forum, 2015. The Travel & Tourism Competitiveness Report 2015.

WTTC. World Travel & Tourism Council. http://www.wttc.org/ (accessed 5.9.16).

WWF. Priority & Endangered Species. http://wwf.panda.org/what_we_do/endangered_species/ (accessed 5.9.16).
